# Immediate application of negative pressure wound therapy following lower extremity flap reconstruction in sixteen patients

**DOI:** 10.1038/s41598-021-00369-5

**Published:** 2021-10-27

**Authors:** Chun-Yu Chen, Shyh-Ming Kuo, Yih-Wen Tarng, Kai-Cheng Lin

**Affiliations:** 1 Department of Orthopaedics, Kaohsiung Veterans General Hospital, 386 Ta-Chung 1st Road, Kaohsiung City, Taiwan, ROC; 2 Department of Occupational Therapy, Shu-Zen Junior College of Medicine and Management, Kaohsiung City, Taiwan, ROC; 3 Department of Biomedical Engineering, I-Shou University, Kaohsiung City, Taiwan, ROC

**Keywords:** Health care, Medical research

## Abstract

Negative pressure wound therapy (NPWT) is usually applied in wound management and soft-tissue salvage after the development of complications. However, immediate postoperative application of NPWT over the flap coverage is seldom reported. We evaluate the effectiveness of immediate postoperative application of NPWT following fasciocutaneous or muscle flap coverage for lower leg reconstruction. A retrospective review of patients who underwent either fasciocutaneous or muscle flap coverage of lower leg soft-tissue defects applied with NPWT immediately after surgery was conducted in a level I trauma center. Sixteen patients, with an average age of 51.2 years, were included in the study. Nine patients had trauma-related soft-tissue loss, six had subsequent soft-tissue defects after debridement, and one had burn injury. Two patients had been treated with free anterolateral thigh flaps, 11 with pedicle flaps, and three with muscle flaps. All flaps survived except for those in two patients with venous congestion on postoperative day 1, which needed further debridement and skin grafting. Therefore, the use of immediate incisional NPWT is an alternative for wound care following flap coverage. The U-shaped design allows easy flap observation and temperature check. Furthermore, this method eliminates any concerns of vascular pedicle compression under negative pressure.

## Introduction

First described by Morykwas et al., negative pressure wound therapy (NPWT) is the use of a gentle vacuum for the management of persistent wounds^1,2^. NPWT can provide cover for wounds under sterile conditions and potentially delay flap coverage until a healthier wound bed is formed. This method promotes wound healing by enhancing the blood supply to the wound bed before flap reconstruction, which is especially beneficial in the case of exposed fractures, highly contaminated wounds, chronic wounds with reduced healing potential, and burns^3^. Therefore, NPWT has been widely used in the treatment of large and chronic wounds, including those of the chest^4–6^, abdomen^7^, extremities^8–10^, pharynx^11^, and head and neck^12^. Moreover, NPWT has also been used to enhance the integration of artificial skin and dermal substitutes and reinforce skin grafts^13,14^.

In most reported cases, NPWT was applied as a staged treatment before soft-tissue coverage. Several recent studies have reported that NPWT can be used to promote salvage when flap survival is in doubt^15,16^. Use of NPWT may resolve venous congestion by promoting local blood flow and venous return from the wound edge to reduce interstitial blood congestion, facilitating revascularization between the transferred flap and the recipient wound bed through neoangiogenesis, and reducing the interstitial space and using pressure to remove excess fluid and infectious material from the wound bed. Since NPWT can improve postoperative venous congestion, which is one of the most common causes of flap failure, immediate postoperative application of NPWT coupled with flap coverage can be considered^17–19^. However, its safety and standard practice of application over flaps immediately after surgery remain unclear.

This study evaluates the effectiveness of immediate postoperative application of NPWT following fasciocutaneous or muscle flap coverage for lower leg reconstruction. It also describes our own standard practice of NPWT application in a case series.

## Methods

A retrospective review was conducted from a prospectively recorded database in a level I trauma center. The Institutional Review Board of Kaohsiung Veterans General Hospital approved this study (VGHKS18-CT1-20). This study was conducted in accordance with the Declaration of Helsinki and its later amendments. Informed consent was obtained from patients for all surgical procedures and wound management, and for the possible use of anonymized photographs. We assessed all patients who underwent either fasciocutaneous or muscle flap coverage of lower leg soft-tissue defects applied with NPWT straight after surgery between January 2016 and February 2019 with at least six months postoperative follow-up. All operations were performed by the same orthopedic surgeon (C-Y, C). Charts were reviewed for patient demographics, comorbidities, cause of defect, flap type, complications, and wound outcomes.

### Immediate application of NPWT

NPWT was immediately applied after fasciocutanous flap coverage and surgical closure had been completed. No drainage tube was inserted underneath the flap. Long strips of gauze dressing impregnated with framycetin sulfate [Sofra-Tulle®, Sanofi Aventis, Paris, France] were placed along the suture line to prevent direct injury and skin maceration and avoid adhesion during the subsequent removal of the foam dressing.

**Figure 1:**
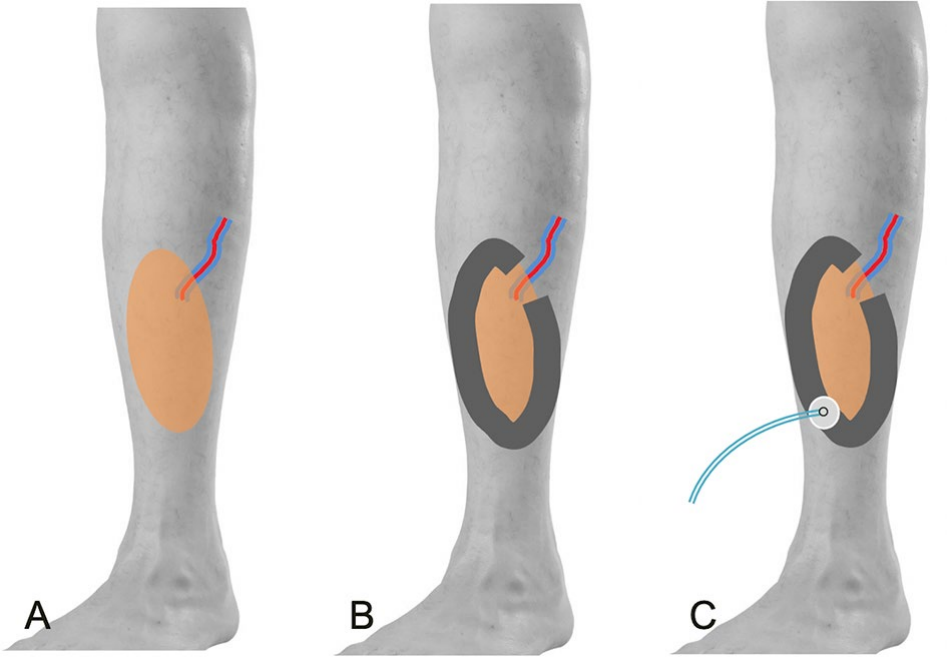
(**A**) The defect was managed with fasciocutaneous flap coverage, and surgical closure had been completed. (**B**) U-shaped foam dressing was applied along the suture line of the flap. The opening of the foam dressing prevents compression over the path of the vessel pedicles to flap. (**C**) The NPWT machine was set with the tubing and suction connector on the opposite side of the vessel pedicles.

A reticulated open cell foam dressing [V.A.C.® Granufoam™ Dressing; KCI, now part of 3 M company, San Antonio, TX, USA] was fashioned into a U shape and then applied directly over the suture line. The opening of the U-shaped foam was placed at the exact path of the vessel pedicle to the flap, so compression over the vessel pedicle could be prevented when negative pressure causes the foam to collapse (Fig. 1). The NPWT machine [INFOV.A.C.™ Therapy Unit, KCI, now part of 3 M company] was set at a negative pressure of 100 mmHg under intermittent suction mode (5 min of negative pressure followed by 2 min without negative pressure).

For muscle flaps, skin grafts were applied in each case immediately following the placement of well-circulated muscle over the defect. A whole gauze dressing impregnated with framycetin sulfate was placed over the skin graft to avoid adhesion. A reticulated open cell foam dressing [V.A.C.® Granufoam Dressing, KCI, now part of 3 M company] was tailored into a round or elliptical shape to match the muscle flap. NPWT was initiated using intermittent negative pressure at − 100 mmHg (5 min of negative pressure and 2 min without negative pressure).

The flap viability was monitored closely, with the staff nurse assessing the temperature, color, and capillary refill every hour. If there is uncertainty in nurse monitoring, the physician will be informed, and additional confirmation will be made. Otherwise, the documented inspection was performed every 8 h by a physician. Furthermore, drainage amounts were recorded daily until the dressings were removed on the fifth postoperative day.

## Results

A total of 16 patients (11 men) were included in the study (Table 1). Patient ages ranged from 23 to 78 years, with an average age of 51.2 years. A history of diabetes mellitus was noted in seven patients, and four patients are current tobacco smokers. Among the patients, nine had trauma such as open fracture Gustilo IIIB or higher, or trauma-related soft-tissue loss of the lower leg and foot that required coverage. Moreover, six patients had infection and subsequent soft-tissue defect after debridement, and one had a burn injury that needed further coverage. Reconstruction sizes were 12 cm^2^–275 cm^2^, with a mean of 51.09 cm^2^. Two patients had been treated with free anterolateral thigh flaps, 11 with pedicle flaps, and three with muscle flaps.

**Table 1:** Patient characteristics and results.

Patients	Gender	Age	Comorbidities	Location of defect	Size (cm^2^)	Flap	Duration of NPWT (day)	Flap result
1	M	42	DM, smoking	Right lateral ankle	32.5	Perforator propellar flap	5	Survival
2	M	56	DM, smoking	Left heel	35	Perforator propellar flap	5	Survival
3	M	72	DM	Right heel	12	Pedicled perforator flap	5	Survival
4	M	29	None	Left dorsal foot	88	Free ALT (1A1V)	5	Survival
5	M	26	None	Left lower leg	22	Perforator propellar flap	5	Survival
6	F	78	DM	Right medial ankle	13.5	Perforator propellar flap	1	Partial flap failure
7	M	46	None	Left lower leg	48	Pedicled perforator flap	5	Survival
8	F	76	None	Left heel	42.5	Reverse sural flap	5	Survival
9	F	34	DM	Right lower leg	275	Free ALT (1A2V)	5	Survival
10	M	40	Smoking	Left lower leg	75	Reverse sural flap	1	Partial flap failure
11	M	70	None	Left heel	35	Reverse sural flap	5	Survival
12	M	26	DM	Left lower leg	12	Perforator propellar flap	5	Survival
13	F	77	None	Right lower leg	20	Hemisoleus muscle flap	5	Survival
14	F	49	DM	Left lower leg	30	Gastrocnemius muscle flap	5	Survival
15	M	23	None	Right heel	35	Reverse sural flap	5	Survival
16	M	75	Smoking	Right lower leg	42	Gastrocnemius muscle flap	5	Survival

*ALT* anterolateral thigh, *DM* diabetes mellitus, *NPWT* negative pressure wound therapy, *A* anastomosed artery, *V* anastomosed vein.

**Figure 2:**
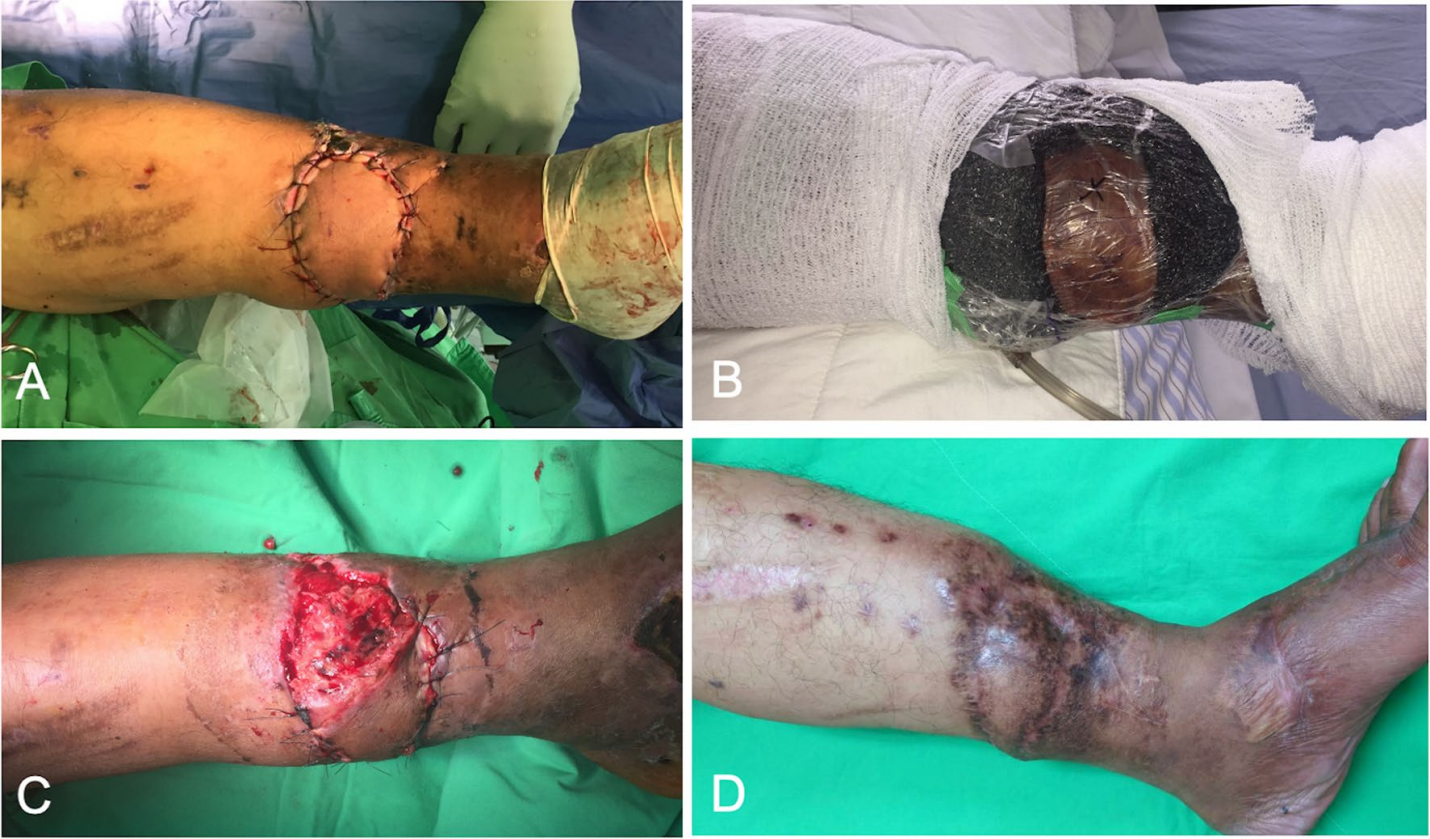
(**A**) A 40-year-old man with a smoking history underwent a reverse sural flap for his left lower leg with osteomyelitis and soft-tissue defects. (**B**) Venous congestion was noted on postoperative day 1, then NPWT was discontinued. (**C**) Partial loss of the distal part of the flaps was subsequently treated with debridement and skin grafting. (**D**) After one year, the wound has completely healed.

Fourteen flaps survived without complications. No occurrence of surgical-site infection was observed. Venous congestion was noted on postoperative day 1 in two patients, who underwent reverse sural flap (Fig. 2) and perforator propeller flap, respectively (Table 1). We discontinued NPWT and removed part of the stitches in these two patients, with local subcutaneous Heparin injection performed along the wound edge of the flap. In the next few days, NPWT was terminated and standard postoperative dressings were changed regularly to monitor the flaps. This resulted in partial loss of the distal part of the flaps, which was subsequently treated with debridement and skin grafting. No other patient developed complications or had compromised status in the postoperative period.

## Case 1

**Figure 3:**
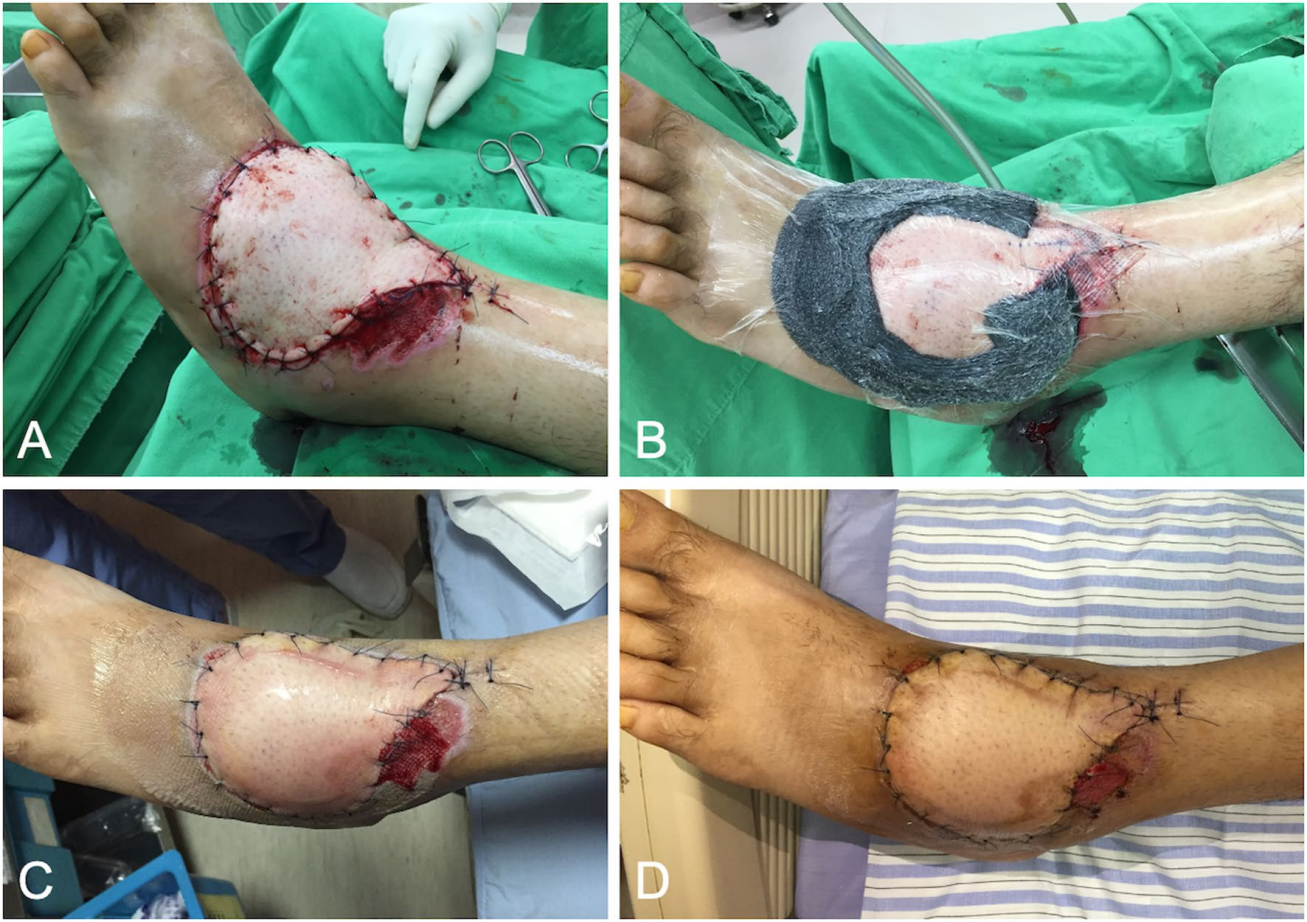
(**A**) An 8 × 11 cm defect over the dorsal foot was covered with free anterolateral thigh flap. (**B**) NPWT was applied immediately following surgery. (**C**) Mild maceration over the flap edge but normal perfusion while discontinuing the NPWT. (**D**) One week after discontinuation of NPWT.

A 29-year-old man presented with an 8 × 11 cm open wound over the left dorsal foot due to a motorcycle accident. The defect included the dorsalis pedis artery, dorsal-lateral ligaments, and capsule, combined with a massive defect of the extensor digitorum longus and extensor digitorum brevis. On admission, debridement was performed several times until the infectious status was controlled. A one-stage operation of free anterolateral thigh free flap was performed with simultaneous extensor digitorum longus reconstruction using the fascia lata. NPWT was applied immediately after flap fixation (Fig. 3). Five days after NPWT, the flap edge showed a small amount of maceration; however, the whole flap exhibited good perfusion status.

## Case 2

**Figure 4:**
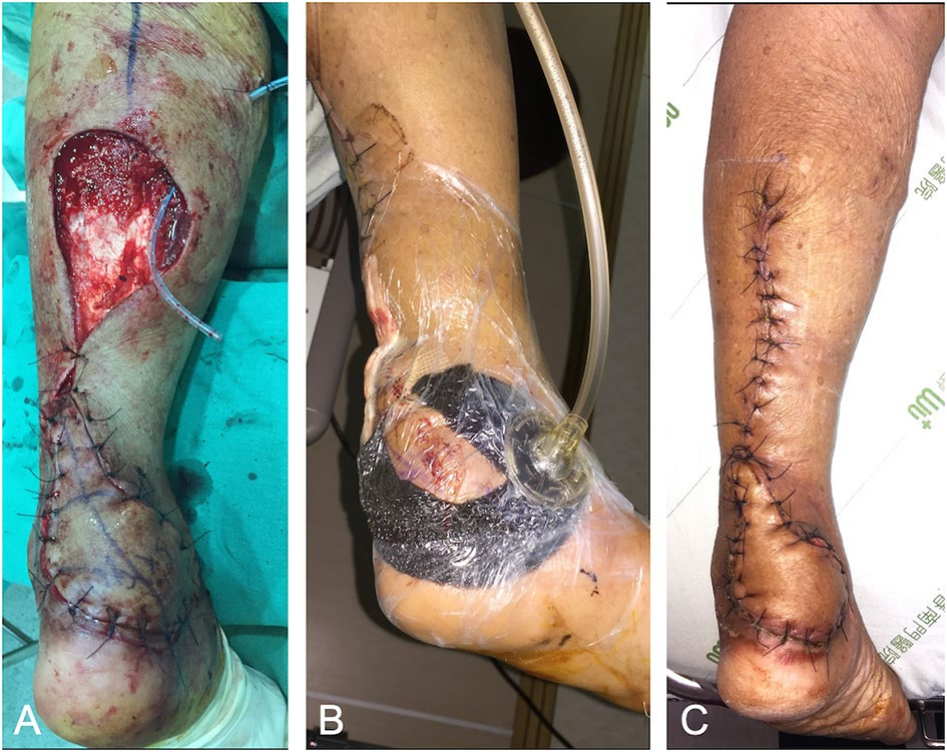
(**A**) A 6 × 8 cm defect over the left heel was covered with reverse sural flap. (**B**) NPWT was applied immediately following surgery, with an opening left to avoid compression of the pedicle from the posterior tibial artery. (**C**) On postoperative day 5, the NPWT was discontinued and the flap showed a well-circulated appearance.

A 76-year-old woman with an exhaust pipe-related burn injury involving the left heel had undergone debridement and presented with a 6 × 8 cm soft-tissue defect. We performed a reverse sural flap for coverage. Because of the reverse flow from the branch of the posterior tibial artery, venous congestion was predicted. We immediately applied NPWT along the suture line and left an opening to prevent compression of the pedicle from the posterior tibial artery (Fig. 4). On postoperative day 5, NPWT was discontinued, and the flap showed a well-circulated appearance and complete survival.

## Discussion

NPWT is traditionally utilized for wound management and soft-tissue salvage after the development of complications. The immediate postoperative application of NPWT after reconstruction using flap coverage is seldom reported. We evaluated the effectiveness of the immediate postoperative application of NPWT following muscle or fasciocutaneous flap coverage for lower leg reconstruction. All flaps survived without complications in 14 of 16 patients following the application of NPWT immediately after reconstruction.

NPWT draws wound edges together, decreases edema in the local tissue, removes exudate and infectious materials from the wound bed, protects and maintains a moist wound environment, and optimizes the blood flow to the wound and promotes neovascularization of the wound bed^8,20–23^. In our practice, we commonly use incisional NPWT over a closed incision in patients with trauma at high risk for wound-related complications, such as patients with calcaneus, distal tibial, and proximal tibial fractures, and diabetes mellitus with poor control^24^.

Published studies also support the use of NPWT following lower extremity trauma. Schlatterer et al. conducted a systematic review of NPWT use in Grade IIIB open tibial fractures and reported lower infection rates with NPWT compared with conventional dressing^25^. Schlatterer et al. also supported NPWT use as an adjunctive modality to reduce flap coverage rates in cases that required soft-tissue procedures^25^. A finding supported by Stannard et al. showed that negative pressure was effective in reducing the infection rate and facilitating wound healing in Grade IIIB fractures^26^.

The immediate postoperative application of NPWT for fasciocutaneous pedicle or free flap has not been extensive due to concerns of the vascular pedicle compression. However, one published study supported the use of NPWT. Lin et al. conducted a retrospective comparative study to assess the difference between NPWT and standard wound care in patients who underwent free flap reconstruction immediately following head and neck cancer surgery and radical neck lymph node dissection^19^. The authors supported the immediate use of NPWT after free flap reconstruction due to reduced rates of complications and survival of all flaps in the NPWT group.

Vascular congestion can also develop after flap reconstruction, potentially leading to flap loss. Venous congestion often occurs due to a kinked perforator of the propeller flap, venous stasis, absence of sizable vein for anastomosis, impaired peripheral microcirculation, and nonphysiological flap reconstruction with a reverse flow. Previous studies have concluded that mechanical stretch by negative pressure promotes the release of vascular endothelial growth factor and regulates new tissue formation^23,27^. Furthermore, some studies have suggested that NPWT may modify the ultrastructure of capillaries and endotheliocytes to support neovascularization and enhance blood flow^28^, resulting in increased flap viability. Vaienti et al. obtained promising results with NPWT on three congested and edematous pedicle flaps and one free flap^29^. The compromised venous return was resolved after NPWT use. Similar results were achieved by Qiu et al., who used NPWT in 12 patients with venous congestion after pedicle or free flap reconstruction^16^, where all congested flaps survived. Venous congestion was resolved by NPWT is likely because of better venous return and tissue oxygen delivery.

Based on our experience of incisional NPWT use, we cut the foam dressing into a U shape and applied it along the suture line after fasciocutaneous flap reconstruction. This left an open area over the pedicle axis, which can prevent compression and allow for monitoring of the flap’s condition and temperature. There are available stripped foam dressings for NPWT designed for incision wounds, which can also be used directly around the flap edge. In our patient cohort, there were two cases of venous congestion that occurred on the first postoperative day. However, we believe that comorbidities were responsible for the development of complication as one patient was a heavy smoker and the other had a history of poorly controlled diabetes mellitus. These two flaps eventually survived after removing part of the incision sutures to allow for decompression and applying anticoagulant agents.

NPWT can also be applied immediately after flap fixation to provide better venous return and promote circulation in the flap. Previous successive studies supported the application of NPWT over cutaneous flaps^30^ and gastrocnemius muscle flaps^31^. Lance et al. analyzed eight patients who underwent pedicled gastrocnemius muscle flap procedures with NPWT to immobilize split-thickness skin graft resulting in the absence of flap necrosis and a 97.5% ± 5.5% mean split-thickness skin graft uptake^31^. In our case series, there were two gastrocnemius muscle flaps and one hemisoleus muscle flap managed with negative pressure. The gastrocnemius muscle flap was nourished via the sural artery, which entered the muscle far from the wound defect and supplied the microcirculation in the entire muscle; therefore, vessel compression was not a concern. We applied foam dressing to cover the entire muscle and immobilize the skin graft. All three muscle flaps survived with a well-attached skin graft.

## Conclusion

The use of immediate NPWT following flap coverage provides reliable and effective management. We recommend setting the vacuum machine at 100 mmHg under intermittent negative pressure and using a U-shaped foam dressing for fasciocutaneous flap and complete foam dressing coverage for muscle flap. The U-shaped design for fasciocutaneous flap reconstruction allows for easy flap observation and monitoring, and eliminates fears of the vascular pedicle compression under negative pressure. This study was limited by its small sample size and lack of control group. A randomized controlled study with a larger sample size is needed to fully assess the effect of immediate application of NPWT after flap reconstruction.
